# Discovery of candidate genes involved in ethylene biosynthesis and signal transduction pathways related to peach bud cold resistance

**DOI:** 10.3389/fgene.2024.1438276

**Published:** 2024-07-18

**Authors:** Wenqian Xia, Yupeng Yang, Chenguang Zhang, Chunsheng Liu, Kun Xiao, Xiao Xiao, Junkai Wu, Yanhong Shen, Libin Zhang, Kai Su

**Affiliations:** ^1^ College of Horticulture Science and Technology, Hebei Normal University of Science and Technology, Qinhuangdao, China; ^2^ Hebei Key Laboratory of Horticultural Germplasm Excavation and Innovative Utilization, Qinhuangdao, China; ^3^ Hebei Higher Institute Application Technology Research and Development Center of Horticultural Plant Biological Breeding, Hebei Normal University of Science and Technology, Qinhuangdao, China

**Keywords:** peach buds, cold resistance, ethylene, transcription factor, regulatory mechanism, candidate gene

## Abstract

**Background:** Low temperature pose significant challenges to peach cultivation, causing severe damage to peach buds and restricting production and distribution. Ethylene, an important phytohormone, plays a critical role in enhancing plant cold resistance. Structural genes and transcription factors involved in ethylene biosynthesis and signal transduction pathways are associated with cold resistance. However, no research has specifically addressed their roles in peach cold resistance.

**Methods:** In this study, we aimed for cold-resistance gene discovery in cold-sensitive peach cultivar “21Shiji” (21SJ) and cold-resistance cultivar “Shijizhixing” (SJZX) using RNA-seq and gas chromatography.

**Results:** The findings revealed that under cold stress conditions, ethylene biosynthesis in “SJZX” was significantly induced. Subsequently, a structural gene, *PpACO1-1*, involved in ethylene biosynthesis in peach buds was significantly upregulated and showed a higher correlation with ethylene release rate. To identify potential transcription factors associated with *PpACO1-1* expression and ethylene signal transduction, weighted gene co-expression network analysis was conducted using RNA-seq data. Four transcription factors: *PpERF2*, *PpNAC078*, *PpWRKY65* and *PpbHLH112*, were identified.

**Conclusion:** These findings provide valuable theoretical insights for investigating the regulatory mechanisms of peach cold resistance and guiding breeding strategies.

## Introduction

Peach (*Prunus persica* L.) originates in China. It is widely cultivated in the North China Plain and the Yangtze River Basin, where it holds the top rank globally in both yield and cultivation areas. However, the low temperature (<0°C) during winter and early spring in Northern China significantly restricts peach growth and geographical distribution. Most peach cultivars are susceptible to the environments, and those with higher cold resistance are insufficient to meet the market demand. This scarcity significantly limits the high-quality development of the peach industry.

Two regulatory pathways are recognized in plant cold resistance research: the abscisic acid (ABA)-dependent and ABA-independent pathway. Among them, the ABA-independent pathway can be further categorized into C-repeat binding transcription factor (*CBF*)-dependent regulatory and CBF-independent regulatory pathways (Park et al., 2015; Lim and Lee, 2020). Multiple studies have reported that ABA signaling significantly induces cold resistance gene expression in plants (Ju et al., 2020; Guo et al., 2021; Lee et al., 2021; Song et al., 2022; Yu et al., 2023). The ABA receptor protein (*PYR*/*PYL*/*RCAR*), 2C protein phosphatase (*PP2C*), and sucrose nonfermenting-1-related protein kinase 2 (*SnRK2*) constitute the primary components of the ABA signal transduction pathway. ABA presence inactivates PP2C and releases *SnRK2*. Activated *SnRK2* then binds to ABA response elements (ABRE) or ABA response binding factors (ABF) in downstream cold gene promoters, thereby activating their expression (Ma et al., 2009; Nakashima et al., 2009; Klingler et al., 2010; Gonzalez-Guzman et al., 2012; Soon et al., 2012). Recent studies indicate that several transcription factors, such as b-ZIPs, LEAs, MYBs, WRKYs, and NACs, can be regulated by ABA signaling under cold stress (Sun et al., 2019; Ju et al., 2020; Shu Y. et al., 2023; Mei et al., 2023; Shen et al., 2023).

CBFs belong to the APETALA2/ETHYLENE RESPONSE FACTOR (AP/ERF) family. In the ABA-independent pathway, CBFs play a central role in regulating plant cold resistance. Under cold stress, the CBFs can be induced by Inducer of CBF Expression (*ICE*). The induced CBFs then bind to the C-repeat/Dehydration Responsive Element (*CRT/DRE*), a *cis*-acting element in the promoter region of Cold Regulated (*COR*) genes, thereby activating their transcription (Thomashow, 1999; Chinnusamy et al., 2007). Several CBFs involved in plant cold resistance have been identified (Park et al., 2015; Jia et al., 2016; Wang H. et al., 2021; Wang et al., 2021). Among these, the *ICE*-*CBFs*-*COR* signaling cascade stands out as a typical cod-resistant pathway, extensively documented in the literature (Hwarari et al., 2022; Kopeć et al., 2022; Ma et al., 2023; Wang et al., 2023). Furthermore, several transcription factors can directly regulate the expression of CBF genes, thereby improving plant cold resistance, such as *MdNAC104*, *MdHYL1*, *MdMYB88*, and *MdMYB124* in apples (Mei et al., 2023; Shen et al., 2023).

In CBF-independent pathways, structural genes and transcription factors involved in ethylene signal transduction are pivotal for cold resistance (Shu P. et al., 2023). Ethylene biosynthesis begins with methionine conversion to S-adenosyl-methionine (*SAM*) by *SAM* synthetases. *SAM* is further converted to the ethylene precursor 1-aminocyclopropane-1-carboxylic acid (*ACC*) by *ACC* synthetases. Ultimately, *ACC* is converted to ethylene by *ACC* oxidases (*ACO*) (Adams and Yang, 1977; Adams and Yang, 1979; Hamilton et al., 1991; Pattyn et al., 2021). The ethylene receptors and CONSTITUTIVE TRIPLE RESPONSE1 (*CTR1*) downstream of ethylene synthesis negatively regulate the ethylene-signaling pathway. The presence of ethylene inactivates the ethylene receptors and *CTR1*, activating ETHYLENE INSENSITIVE 2 (*EIN2*) expression. *EIN2*, downstream of *CTR1*, promotes the activity of ETHYLENE INSENSITIVE 3 (*EIN3*) and EIN3-LIKE 1 (*EIL1*) (Chen et al., 2010; Shakeel et al., 2015; Zhao et al., 2021), which controls the expression of numerous ethylene-responsive genes, including ETHYLENE RESPONSE FACTORs (ERFs) (Lorenzo et al., 2003; Cheng et al., 2013; Li et al., 2019; Hu et al., 2020). In plants, ERFs contribute positively to cold resistance. For instance, in apples, overexpressing MdERF1B enhances cold tolerance by interacting with *MdACO1* and *MdERF3*, key components in ethylene biosynthesis (Wang et al., 2021a). In *Vitis amurensis*, overexpressing the *VaERF092* gene in the ethylene signal transduction pathway enhances cold resistance of Arabidopsis. Additionally, *VaERF092* interacts with the cis-acting element (GCC-box) in the *VaWRKY33* promoter, indirectly enhancing the cold resistance of Arabidopsis (Sun et al., 2019). In bermudagrass, *CdERF1* positively regulates plant cold response by activating the expression of *PODs*, *CBF2* and *LTPs* (Hu et al., 2020). *ERF41* and *ERF180* in kiwi fruit are significantly induced under low temperatures (Gunaseelan et al., 2019).

Several studies have explored the chilling response of postharvest peach fruit and the cold response of peach shoots (Pons et al., 2014; Wang et al., 2017; Yu et al., 2020; Guo et al., 2023; Li et al., 2023). Peach buds are notably more susceptible to cold stress compared to the trunk and shoots. In Northern China, prolonged winter low temperatures can lead to peach bud ossification, significantly reducing peach orchard yield. However, their studies regarding the cold resistance of peach buds remain unexplored. Therefore, in this study, we aimed to investigate the essential structural genes and transcription factors associated with resistance in peach buds using the cold-sensitive peach cultivar “21Shiji” (21SJ) and the cold-resistant cultivar “Shijizhixing” (SJZX), commonly cultivated in Northern China. “21SJ” came from the hybridization of peach cultivar “Dangui” × “Xuetao” and “SJZX” came from the hybridization of “21Shiji” × “Jiucui”. The approach involves transcriptome analysis and weighted gene co-expression network analysis (WGCNA). The findings could serve as a valuable reference for future research on cold resistance research in peach buds.

## Materials and methods

### Plant material

Peach cultivars “21SJ” and “SJZX” were cultivated in the Peach Experimental Garden of Hebei Normal University of Science and Technology (39°42′N, 119°10′E). Dormant buds from “21SJ” and “SJZX” were utilized to identify cold resistance. Peach shoots (1 year old) containing dormant buds were randomly collected in 20 November 2022, the temperature at that time was ranged from 1°C to 5°C. The collected buds were then subjected to cold storage in a programmable incubator set at −4°C for 0, 12, 24, 48, and 72 h. For ethylene release rates assessment, dormant bud samples of these two cultivars were collected and randomly assigned to 5 sets of 10 buds each (in total 100 buds per time point). Dormant bud samples which used for RNA-seq were randomly collected at 12, 24, 48 h, 3 sets of 10 buds were used at each time point.

### Determination of electrolyte leakage and ethylene release rate

The full buds were excised from peach shoots using flat cuts and subsequently placed in a dish with moist blotting paper and cut into small pieces. Approximately 0.2 g of each sample was incubated in 30 mL of ddH_2_O for 2 h at 25°C with shaking at 200 rpm. The first electrolyte (C1) and second electrolyte leakage were measured using a digital conductivity meter (DDS−307, Rex, China). C2 was achieved after boiling the bud samples at 100°C for 30 min and subsequently cooled down to 25°C with shaking. Relative electrolyte leakage (REL) was calculated as (C1/C2) × 100%. 10 buds of each set were sampled and placed in sealed tube (5 mL), 1 mL of air from the headspace of each tube was withdrawn with a syringe and manually injected into a gas chromatograph (7890A, Agilent Technology, United States). The ethylene release rate in the buds was determined according to the method described by Tian et al. (2013).

### Analysis of peach bud transcriptomics at different cold stress times

RNA concentration and purity were determined using the NanoDrop 2000 (Thermo Fisher Scientific, Wilmington, DE, United States). RNA integrity was assessed with the RNA Nano 6000 Assay Kit on the Agilent Bioanalyzer 2100 system (Agilent Technologies, CA, United States). Sequencing libraries were prepared with 1 μg RNA per sample using the NEBNext Ultra TM RNA Library Prep Kit for Illumina (NEB, United States) following the instructions of the manufacturer. The prepared libraries were sequenced on an Illumina platform, yielding clean reads after adapter, poly-N sequence, and low-quality read removal. The high-quality, paired-end clean reads from each sample were aligned to the Prunus_persica.Chinese_Cling_v1.0.genome [Prunus persica genome assembly ASM1834083v1 - NCBI - NLM (nih.gov)] using Hisat2 software. Subsequently, differential gene expression analysis was performed using DESeq2.

### Identification of candidate genes using qRT-PCR

Total RNA was extracted from “21SJ” and “SJZX” bud samples at 0, 12, 24, 48, and 72 h using the Plant Total RNA Isolation Kit (SK8631; Sangon Biotech, Shanghai, China), following the instruction of the manufacturer. Subsequently, cDNA synthesis was conducted using the PrimeScript™ RT-PCR Kit (RR047A; TaKaRa Bio, Kusatsu, Japan) and diluted fivefold. Quantitative real-time PCR (qRT-PCR) was performed using ABI QuantStudio™ 6 Flex System (Applied Biosystems). Gene expression levels were calculated using the 2^−^ΔΔ^CT^ method, with three biological replicates per reaction. Primer sequences for the candidate genes are provided in Supplementary Table S5.

## Results

### Cold resistance and ethylene release rate assessment in “21SJ” and “SJZX” buds

**FIGURE 1:**
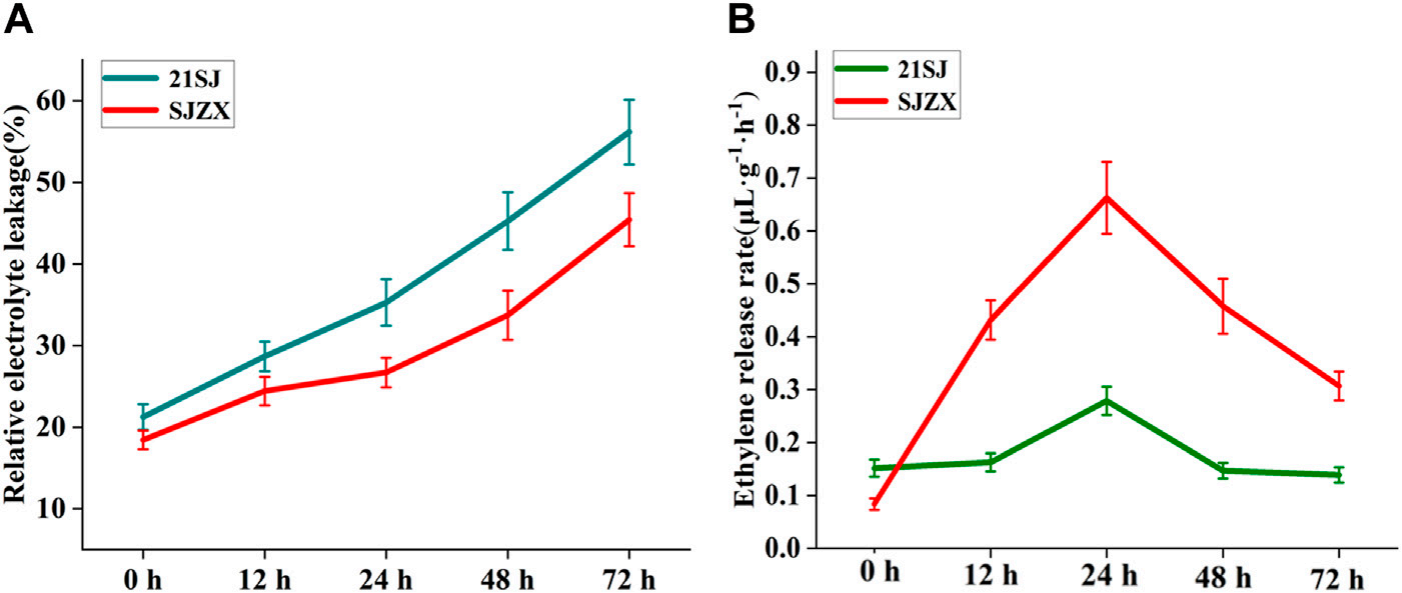
Cold resistance identification and ethylene release rate determination. **(A)** Electrolyte leakage rates determination for “21SJ” and “SJZX” buds at different time. **(B)** Ethylene release rate determination for “21SJ” and “SJZX” buds at different time.

The cold resistance of “21SJ” and “SJZX” buds was measured at different time points under stress using their electrolyte leakage rate (ELR) (Figure 1A). Both cultivars showed increased ELR under cold stress conditions. “21SJ” exhibited an increase from 21.25% to 56.17%, while “SJZX” increased from 18.43% to 45.42%. “21SJ” consistently had significantly higher ELR values compared to “SJZX” from 12 h to 72 h. No significant difference in ELR between “SJZX” at 12 h and 24 h was observed.

To investigate the relationship between ethylene biosynthesis and the different cold resistance of “21SJ” and “SJZX”, ELR was analyzed in these cultivars at different stages of cold stress using gas chromatography (Figure 1B). The findings showed that both cultivars experienced an increase in ELR from 0 h to 24 h during cold stress, reaching a peak at 24 h, followed by a decrease from 24 h to 72 h “SJZX” exhibited a significantly higher ELR compared to “21SJ” from 12 h to 72 h, suggesting a strong link between ethylene biosynthesis and cold resistance in peach buds.

### Differential gene expression analysis between “21SJ” and “SJZX” using transcriptomics

**FIGURE 2:**
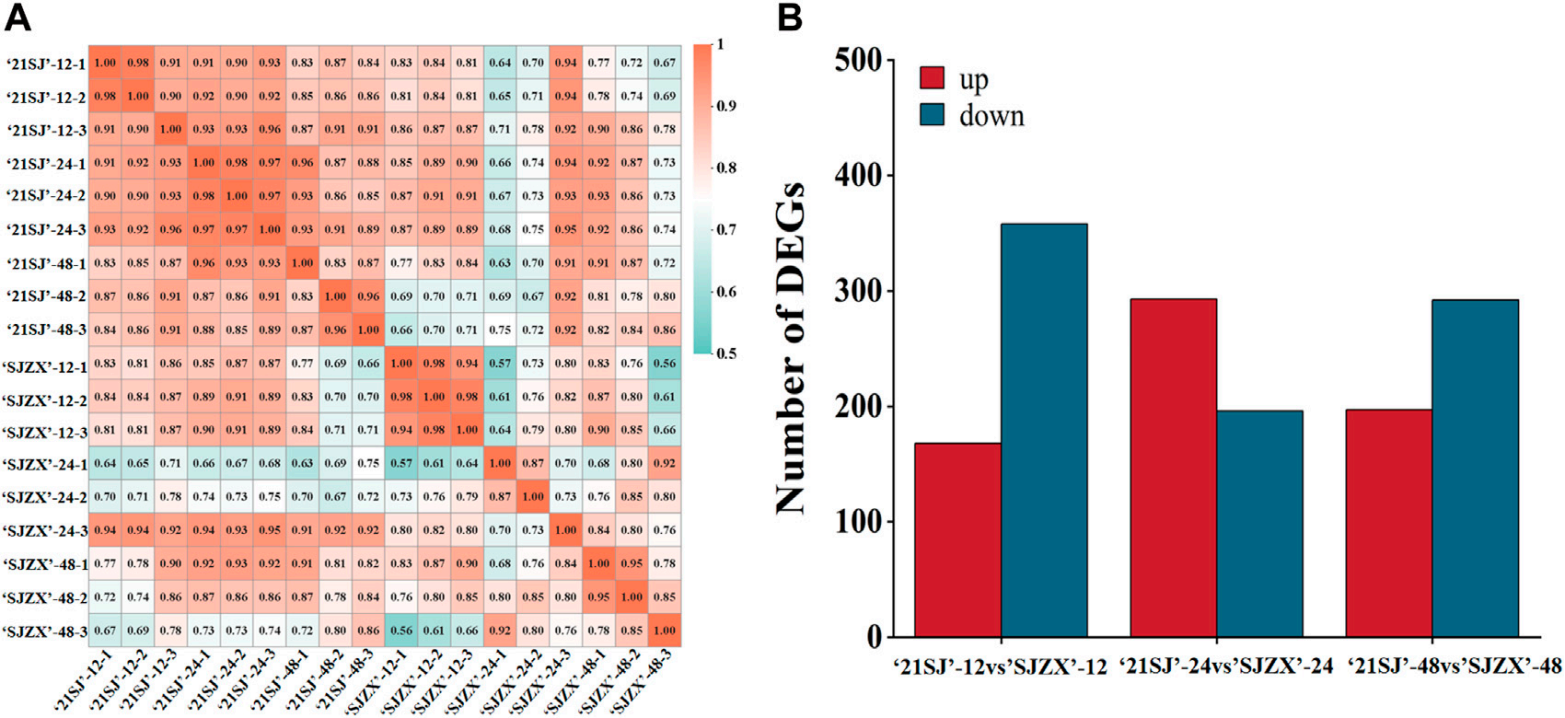
Pearson’s correlation coefficients analysis for all expressed genes and different expressed genes statistics between each group. **(A)** reliability and rationality between samples based on Pearson’s correlation coefficients for all gene expression levels between each sample. **(B)** Values plotted in red represent up-regulated genes in “21SJ” vs. “SJZX”; those plotted in blue represent down-regulated genes in “21SJ” vs. “SJZX” and red plot represent up-regulated genes in “21SJ” vs. “SJZX”.

In this study, peach buds subjected to cold stress treatments for 12, 24, and 48 h underwent RNA-Seq to identify candidate genes. After filtering out low-quality raw reads, a total of 123.91 Gb clean data were obtained and deposited in the NCBI Sequence Read Archive (SRA) with accession number PRJNA1071065 and Q30 (%) was 93.58%–95.29% (Supplementary Table S1). These clean reads were aligned to the reference genome sequence using Hisat2 tools, resulting in the identification of 27,506 annotated unigenes through alignment with Nr, eggNOG, KOG, COG, Swiss-Prot, GO, KEGG, and Pfam databases (Table 1; Supplementary Table S2), including 1,807 novel genes (Supplementary Table S3). Expression levels of each unigene are presented in Supplementary Table S4. Correlation coefficients were calculated to assess gene expression consistency (Figure 2A). After this, differential expression analysis revealed significant differences: 526 genes were differentially expressed in 21SJ-12 vs. SJZX-12 (|[log_2_^(fold change)^]| >1 and adjusted *p* < 0.05), with 168 upregulated and 358 downregulated genes. Moreover, 489 genes displayed differential expression in 21SJ-24 vs. SJZX-24, including 293 upregulated and 196 downregulated genes. Furthermore, 489 genes exhibited differential expression in 21SJ-48 vs. SJZX-48, with 197 upregulated and 292 downregulated genes (Figure 2B).

**TABLE 1:** Summary of transcripts annotated in different database.

Database	Annotated gene number	New annotated gene number
NR	27,469	1786
eggNOG	21,546	1,061
KOG	13,216	523
COG	7,999	248
Swiss-Prot	17,827	726
GO	21,760	1,192
KEGG	17,760	868
Pfam	20,416	866

### Discovery of candidate structural genes in ethylene signal transduction

**FIGURE 3:**
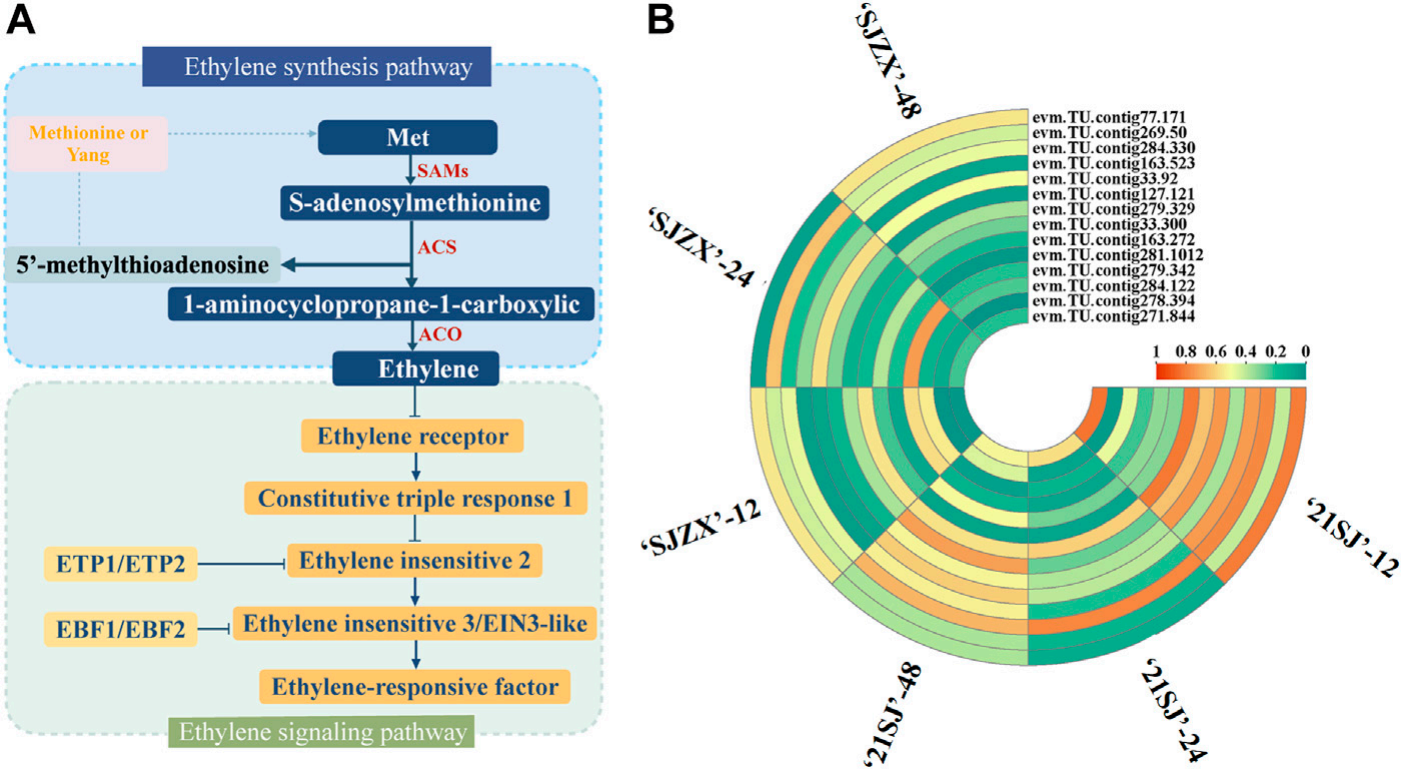
Candidate structural genes in ethylene biosynthesis and signal transduction pathway. **(A)** Structural genes involved in ethylene biosynthesis and signal transduction pathway in plants. **(B)** Candidate genes filtration in ethylene biosynthesis and signal transduction pathway based on RNA-seq data. Orange corresponds to highly expressed and blue to poorly expressed.

Ethylene biosynthesis and signal transduction in plants are complex processes involving several structural genes (Figure 3A). 14 candidate genes were identified from the RNA-Seq data based on their annotation and expression levels in 21SJ-12 vs. SJZX-12, 21SJ-24 vs. SJZX-24, and 21SJ-48 vs. SJZX-48 (Figure 3B).

**FIGURE 4:**
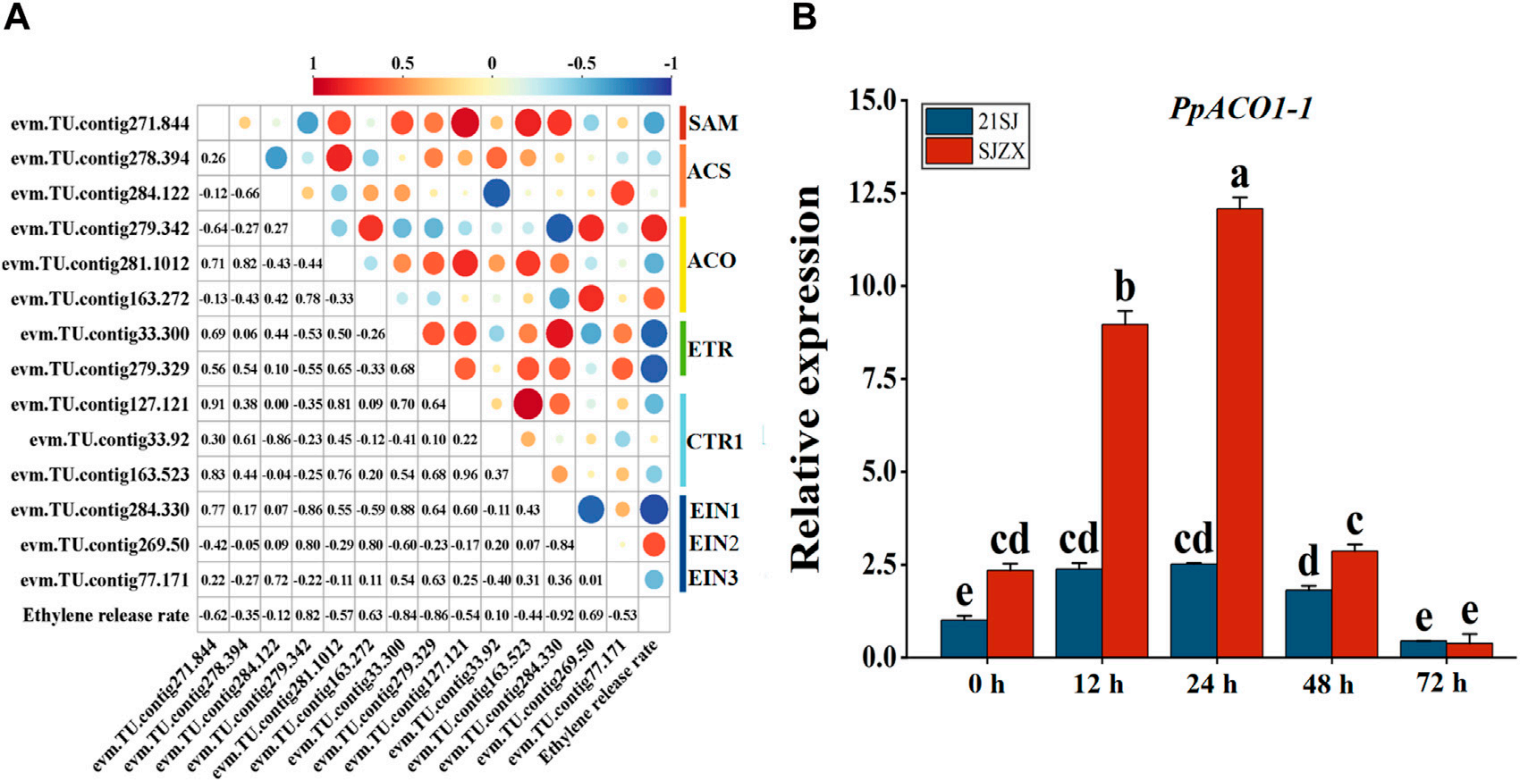
Candidate structural genes discovery and qRT-PCR verification based on Pearson correlation coefficients analysis. Blue bars represent cultivar “21SJ” and red bars represent cultivar “21SJ”. **(A)** Pearson correlation coefficients analysis for candidate genes and ELR. **(B)** qRT-PCR verification for PpACO1-1. Error bars represent the standard deviation of three biological replicates. Lowercase letters on the bar chart represent significant differences between the two cultivars at different cold stress stages according to Duncan’s multiple range test at *p* < 0.05.

To further identify candidate genes associated with ethylene biosynthesis and signal transduction during cold stress in peach buds, Pearson correlation coefficients (*p* < 0.05) were calculated for these 14 candidate genes and ELR (Figure 4A). Subsequently, one structural gene significantly correlated with ELR was identified and designated as *PpACO1-1* (evm.TU.contig279.342). The expression of *PpACO1-1* was significantly induced under cold stress in “SJZX”, peaking at 24 h. Moreover, the expression level of *PpACO1-1* was significantly higher in “SJZX” compared to “21SJ” (Figure 4B).

**FIGURE 5:**
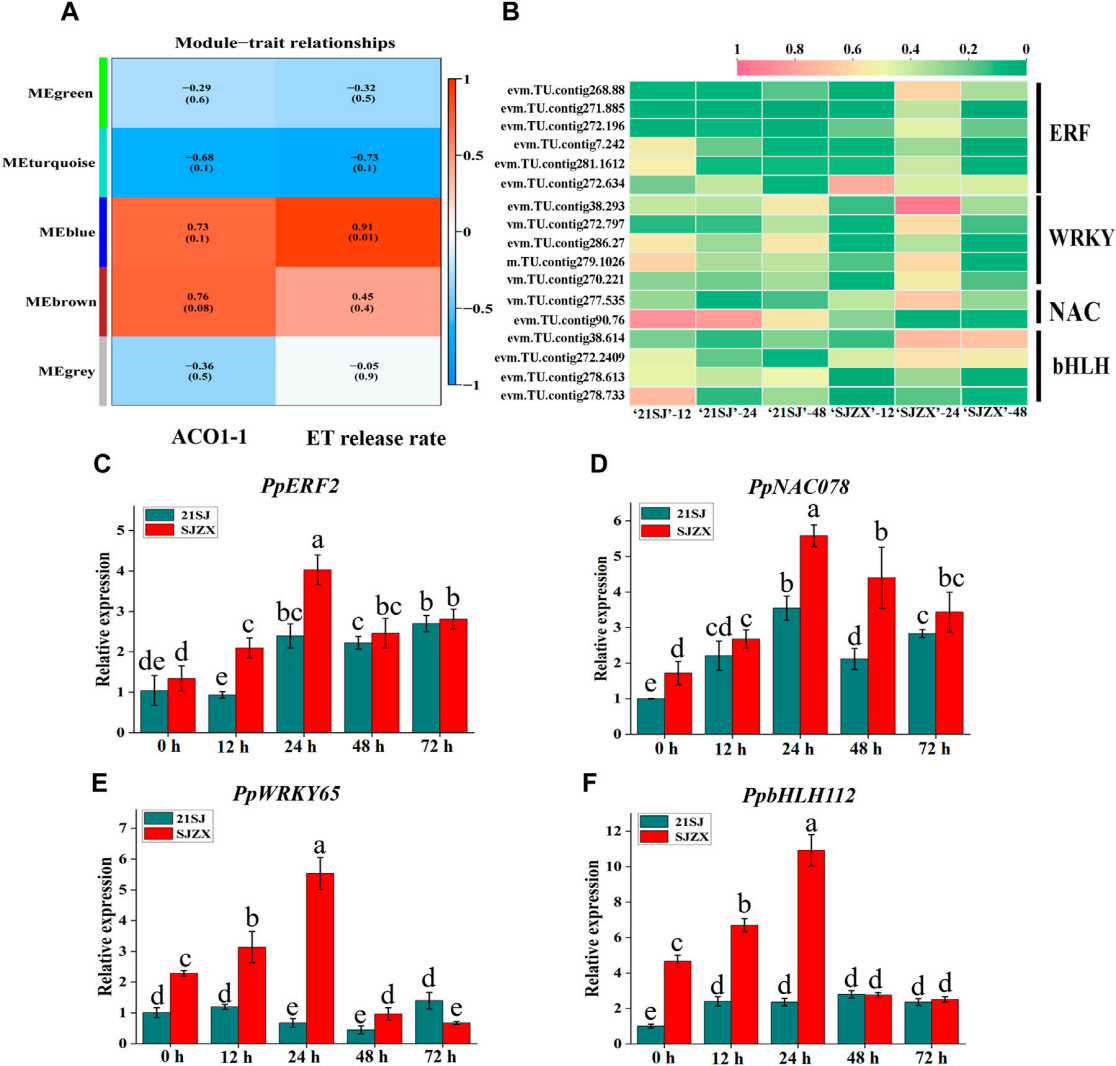
Transcription factors discovery related to peach buds cold resistance based on weighted gene co-expression network analysis and qRT-PCR validation. **(A)** Weighted gene co-expression network analysis based on the expression of *PpACO1-1* and ethylene release rate. **(B)** Cluster heat map of differentially expressed transcription factors in ERF, WRKY, NAC and bHLH family. Genes are shown horizontally; samples are represented by columns. Red corresponds to highly expressed genes and green to poorly expressed genes. **(C**–**F)** qRT-PCR analysis of selected transcription factors involved in peach buds cold resistance. Blue bars represent cultivar “21SJ” and red bars represent cultivar “SJZX”. Error bars represent the standard deviation of three biological replicates. Lowercase letters on the bar chart represent significant differences between the two cultivars and different developmental stages according to Duncan’s multiple range test at *p* < 0.05.

Transcription factors influencing ethylene biosynthesis and the *PpACO1-1* gene were identified using WGCNA. Different modules represented clusters of genes with high correlation (Figure 5A). Initially, 17 transcription factors linked to ERFs, WRKYs, NACs, and bHLHs were identified (Figure 5B). Subsequently, four candidate transcription factors *PpERF2* (evm.TU.contig268.88), *PpNAC078* (evm.TU.contig277.535), *PpWRKY65* (evm.TU.contig38.293), and *PpbHLH112* (evm.TU.contig38.614) were selected based on qRT-PCR (Figures 5C–F). *PpERF2*, *PpNAC078*, and *PpbHLH112* exhibited upregulation in both “21SJ” and “SJZX” peach buds under cold stress, with significantly higher expression levels in “SJZX” than in “21SJ”. Furthermore, *PpWRKY65* showed upregulation in “SJZX” and downregulation in “21SJ,” with its expression level significantly higher in “SJZX” than in “21SJ”.

## Discussion

### Relationship between ethylene biosynthesis and plant cold resistance

Ethylene, an important phytohormone, influences plant growth and development, especially in fruit ripening (McMurchie et al., 1972; Iqbal et al., 2017). Low temperatures causing freezing stress significantly affect plant distribution, growth, and yield, including in peach cultivation (Li and Wang, 2020). Several studies have explored the deep mechanisms of plant resistance to cold stress (Ding et al., 2020; Hwarari et al., 2022; Ma et al., 2022). Phytohormones such as ABA, ET, Jasmonic Acid (JA), and Salicylic acid (SA) are pivotal in plant cold resistance (Huang et al., 2017; Yang et al., 2019; Huang et al., 2023; Zhang et al., 2023). Studies have also highlighted their role in peach cold resistance, including ABA, JA, and SA (Zhang et al., 2009; Zhao et al., 2021b; Zhao et al., 2021c). Ethylene enhances cold resistance in various fruit tree species, such as apples, pears, and grapevine (Hershkovitz et al., 2009; Sun et al., 2016; Wang Y. et al., 2021). However, until now, no research has investigated the relationship between ethylene biosynthesis and cold resistance in peaches. The findings in this study identified a positive correlation between ethylene biosynthesis and cold resistance in peach buds.

Plants synthesize ethylene in response to various biotic and abiotic stresses, either inducing or repressing structural genes in the ethylene biosynthesis and signal transduction pathway (Chen et al., 2009; Boutrot et al., 2010; Xu et al., 2019; Zhao Z. X. et al., 2021; Hartman et al., 2021; Wei et al., 2022). Structural genes such as *SlACS1A*, *SlACS1B*, *SlACO1*, and *SlACO4* in tomato (Dong et al., 2022), *PaACS1*, *PaACS2*, *PaACO*, and *PaCTR1* in pear (Hershkovitz et al., 2009), and *VvACO* and *MdACO1* in apple and grapevine (Sun et al., 2016; Wang Y. et al., 2021), are known for their roles in ethylene biosynthesis under cold stress. However, the key structural gene involved in peach ethylene biosynthesis under cold stress remains unreported. The findings of this study reveal *PpACO1-1* (evm.TU.contig279.342) as a key structural gene responding significantly to cold stress in peach buds.

### Transcription factors involved in ethylene signaling pathway associated with cold resistance

Extensive literature exists on ethylene signaling pathway genes and transcription factors associated with cold resistance compared to the genes involved in ethylene biosynthesis. *ERFs*, important transcription factors regulated by ET, play a significant role in plant cold resistance. For instance, *PtrERF108* in trifoliate orange regulates raffinose synthesis by modulating *PtrRafS* expression, *PtrERF109* positively regulates POD-encoding genes to scavenge reactive oxygen species (ROS), and *PtrERF9* positively modulates ROS homeostasis by regulating *PtrGSTU17* expression under cold stress (Wang M. et al., 2019; Khan et al., 2021; Zhang et al., 2022). Additionally, *ThERF5*, *ThERF31*, *ThERF46*, and *ThERF55* in *Tetrastigma hemsleyanum* exhibit a sensitive response to cold stress (Xie et al., 2022). Furthermore, *MfERF1* from *Medicago falcata* enhances cold tolerance through upregulation of polyamine turnover, antioxidant protection, and proline accumulation (Zhuo et al., 2018). *MdERF1B* in apples enhances cold tolerance by upregulating the expression of the cold-responsive gene *MdCBF1* and ethylene biosynthesis gene *MdACO1* (Wang Y. et al., 2021). *VaERF092* in Amur grape induces cold tolerance calli by regulating *VaWRKY33* expression (Sun et al., 2019). The investigation in this study identified an ERF designated *PpERF2*, which exhibits significantly induced expression in the buds of the cold-resistant cultivar “SJZX”, suggesting its potential function in peach cold resistance.

### Transcription factors involved in CBF-COR cold resistance pathway

NACs, WRKYs, and bHLHs represent three major TF families in plants, acting as key regulators that transmit upstream stress signals to downstream stress responses. Transcription factors from these families are primarily involved in the CBF-COR cold resistance pathway, directly binding to the promoter regions of CBFs to enhance their expression. For instance, *GmNAC20* in soybean (Hao et al., 2011), *PbeNAC1* in pear (Jin et al., 2017), and *MdNAC104* in apple (Mei et al., 2023), *MdCIbHLH1*/*MdICE1*, *MdICE1L* and *MdbHLH4* in apple (Feng et al., 2012; An et al., 2021; An et al., 2022; Yang et al., 2023), *PavbHLH106* and *PavbHLH28* in sweet cherry (Cao et al., 2023; Hou et al., 2023), *VaWRKY33* in grapevine (Sun et al., 2019), *VbWRKY32* in *Verbena bonariensis* (Wang M. Q. et al., 2019), *KoWRKY40* in *Kandelia obovate* (Fei et al., 2022), *CdWRKY2* in bermudagrass (Huang et al., 2022). To date, no research has reported candidate transcription factors from these families involved in peach cold resistance. Three transcription factors, *PpNAC078*, *PpWRKY65*, and *PpbHLH112*, were preliminarily identified in this study. However, further investigation is needed to understand the precise cold resistance mechanisms mediated by these transcription factors.

## Conclusion

In the study, cold-sensitive peach cultivar “21SJ” and cold-resistance cultivar “SJZX” were used to discovering peach bud cold resistant genes by using RNA-seq and gas chromatography. In total of 123.91 Gb clean data were achieved based on RNA-seq and 526, 489 and 489 genes were differentially expressed in 21SJ-12 vs. SJZX-12, 21SJ-24 vs. SJZX-24 and 21SJ-48 vs. SJZX-48, respectively. Finally, the ethylene biosynthesis gene, *PpACO1-1*, was discovered as pivotal in peach bud cold resistance, given its significant response to cold stress in resistant cultivars. Subsequently, four transcription factors *PpERF2*, *PpNAC078*, *PpWRKY65*, and *PpbHLH112* were selected based on WGCNA, as they potentially regulate *PpACO1-1* expression and ethylene biosynthesis. These findings provide crucial insights for future research and breeding endeavors aimed at bolstering peach cold resistance.

## Data Availability

The datasets presented in this study can be found in online repositories. The names of the repository/repositories and accession number(s) can be found in the article/Supplementary Material.
